# Safety and efficacy of dendritic cell-based immunotherapy DCVAC/OvCa added to first-line chemotherapy (carboplatin plus paclitaxel) for epithelial ovarian cancer: a phase 2, open-label, multicenter, randomized trial

**DOI:** 10.1136/jitc-2021-003190

**Published:** 2022-01-06

**Authors:** Lukas Rob, David Cibula, Pawel Knapp, Peter Mallmann, Jaroslav Klat, Lubos Minar, Pavel Bartos, Josef Chovanec, Petr Valha, Marek Pluta, Zdenek Novotny, Jiri Spacek, Bohuslav Melichar, Dariusz Kieszko, Jitka Fucikova, Tereza Hrnciarova, Roman Pawel Korolkiewicz, Marek Hraska, Jirina Bartunkova, Radek Spisek

**Affiliations:** 1 Third Faculty of Medicine, Charles University and University Hospital Kralovske Vinohrady, Prague, Czech Republic; 2 First Faculty of Medicine, Charles University and General University Hospital in Prague, Prague, Czech Republic; 3 Department of Gynaecologic Oncology, Medical University of Bialystok, Bialystok, Poland; 4 University Hospital of Cologne, Cologne, Germany; 5 Department of Gynecology and Obstetrics, University Hospital Ostrava and University of Ostrava, Ostrava, Czech Republic; 6 Department of Gynecology and Obstetrics, University Hospital Brno and Masaryk University, Brno, Czech Republic; 7 Department of Gynecology and Obstetrics, Hospital Novy Jicin Novy Jicin, Novy Jicin, Czech Republic; 8 Masaryk Memorial Cancer Institute, Brno, Czech Republic; 9 Department of Gynecology and Obstetrics, Hospital Ceske Budejovice, České Budějovice, Czech Republic; 10 Department of Obstetrics and Gynecology, 2nd Faculty of Medicine, University Hospital Motol, Prague, Czech Republic; 11 Department of Gynecology and Obstetrics, Faculty Hospital Plzen, Plzen, Czech Republic; 12 Department of Obstetrics and Gynecology, University Hospital Hradec Kralove, Hradec Kralove, Czech Republic; 13 Department of Oncology, Palacky University Medical School and Teaching Hospital, Olomouc, Czech Republic; 14 Oncological Center of the Lublin Region, Lublin, Poland; 15 Department of Immunology, Charles University, Praha, Czech Republic; 16 SOTIO a.s, Prague, Czech Republic

**Keywords:** immunotherapy, clinical trials, phase II as topic, dendritic cells

## Abstract

**Background:**

Most patients with epithelial ovarian cancer (EOC) relapse despite primary debulking surgery and chemotherapy (CT). Autologous dendritic cell immunotherapy (DCVAC) can present tumor antigens to elicit a durable immune response. We hypothesized that adding parallel or sequential DCVAC to CT stimulates antitumor immunity and improves clinical outcomes in patients with EOC. Based on the interim results of sequential DCVAC/OvCa administration and to accommodate the increased interest in maintenance treatment in EOC, the trial was amended by adding Part 2.

**Methods:**

Patients with International Federation of Gynecology and Obstetrics stage III EOC (serous, endometrioid, or mucinous), who underwent cytoreductive surgery up to 3 weeks prior to randomization and were scheduled for first-line platinum-based CT were eligible. Patients, stratified by tumor residuum (0 or <1 cm), were randomized (1:1:1) to DCVAC/OvCa parallel to CT (Group A), DCVAC/OvCa sequential to CT (Group B), or CT alone (Group C) in Part 1, and to Groups B and C in Part 2. Autologous dendritic cells for DCVAC were differentiated from patients’ CD14^+^ monocytes, pulsed with two allogenic OvCa cell lines (SK-OV-3, OV-90), and matured in the presence of polyinosinic:polycytidylic acid. We report the safety outcomes (safety analysis set, Parts 1 and 2 combined) along with the primary (progression-free survival (PFS)) and secondary (overall survival (OS)) efficacy endpoints. Efficacy endpoints were assessed in the modified intention-to-treat (mITT) analysis set in Part 1.

**Results:**

Between November 2013 and March 2016, 99 patients were randomized. The mITT (Part 1) comprised 31, 29, and 30 patients in Groups A, B, and C, respectively. Baseline characteristics and DCVAC/OvCa exposure were comparable across the treatment arms. DCVAC/OvCa showed a good safety profile with treatment-emergent adverse events related to DCVAC/OvCa in 2 of 34 patients (5.9%) in Group A and 2 of 53 patients (3.8%) in Group B. Median PFS was 20.3, not reached, and 21.4 months in Groups A, B, and C, respectively. The HR (95% CI) for Group A versus Group C was 0.98 (0.48 to 2.00; p=0.9483) and the HR for Group B versus Group C was 0.39 (0.16 to 0.96; p=0.0336). This was accompanied by a non-significant trend of improved OS in Groups A and B. Median OS was not reached in any group after a median follow-up of 66 months (34% of events).

**Conclusions:**

DCVAC/OvCa and leukapheresis was not associated with significant safety concerns in this trial. DCVAC/OvCa sequential to CT was associated with a statistically significant improvement in PFS in patients undergoing first-line treatment of EOC.

**Trial registration number:**

NCT02107937, EudraCT2010-021462-30.

## Introduction

Recent estimates suggest that over 300,000 new cases of ovarian cancer are diagnosed annually, accounting for 3.4% of new cancers and 4.7% of cancer-related deaths among women in 2020.^1^ This high mortality rate is driven by the late diagnosis, because early-stage ovarian cancers are usually asymptomatic or are accompanied by non-specific signs and symptoms.^2^

Cancer immunotherapy aiming to exploit the patient immune system for the control of tumor growth has gained significant attention during the last decade. Dendritic cells (DCs) are a vital component of the immune system that internalize and process antigens for presentation and activation of T lymphocytes.^3 4^ DCs may be primed by exposure to a source of tumor-associated antigens (TAAs), and hence be used to activate T lymphocytes capable of targeting cancer cells in vivo.^4^ On this basis, the use of DC-based vaccines for cancer has been extensively investigated, with more than 200 completed clinical studies to date.^4^ This therapeutic strategy involves the isolation or in vitro generation of autologous DCs followed by ex vivo manipulation and reinfusion into patients.^5–10^ These studies of DC-based vaccines predominantly involved melanoma, prostate cancer, glioblastoma and ovarian carcinoma. An autologous active cellular immunotherapy consisting of dendritic cells for ovarian cancer (DCVAC/OvCa) was developed in which allogeneic tumor cells killed by high hydrostatic pressure are used as a source of multiple TAAs for loading onto autologous DCs.^11 12^ Following administration, the prepared DCs elicit a polyclonal T-cell response capable of targeting the existing malignant cells. This approach is expected to increase the therapeutic potency and reduce the likelihood of immune evasion occurring through antigen loss.^13^ DC-based immunotherapy has been tested in a variety of cancers, including prostate cancer^14–16^ and glioblastoma.^13^

To explore the clinical potential of DC therapy in epithelial (EOC) ovarian cancer (DCVAC/OvCa), a first in human, phase I trial of DCVAC/OvCa provided evidence that it elicited an immune response against relevant tumor antigens in patients with stage III–IV EOC, following primary cytoreduction surgery and at least one cycle of chemotherapy (CT).^17^ Following the results of that trial, we implemented an exploratory phase II trial, which is still ongoing (collection of overall survival (OS) follow-up information), to investigate the safety and efficacy of DCVAC/OvCa in patients with EOC who had undergone optimal cytoreductive surgery. The trial initially comprised three groups, in which patients received DCVAC/OvCa in parallel (ie, concomitantly) (Group A) or sequentially (Group B) with platinum-based CT, or CT alone (Group C). The trial explored two alternative schedules of DCVAC/OvCa-carboplatin-paclitaxel front line chemoimmunotherapy to characterize schedule-dependent interactions and explore whether a particular schedule provides enhanced activity of the combination. Because the first interim analysis suggested a progression-free survival (PFS) benefit of DCVAC/OvCa sequential to CT (ie, Group B), it was decided to expand Groups B and C to further explore the efficacy and safety of longer exposure to DCVAC/OvCa sequential to CT.

## Methods

### Ethics and oversight

The trial was conducted in accordance with the Declaration of Helsinki and International Council for Harmonisation guidelines on Good Clinical Practice. The protocol with amendments and the consent form were approved by ethics committees at each participating site. The trial was registered on EudraCT and ClinicalTrials.gov. Trial oversight was provided by the steering committee and data were reviewed by an independent data monitoring committee, which provided recommendations on whether the trial could continue or should be terminated. The first patient was enrolled in November 2013. The data export for the current analysis was performed in November 2020.

### Patients

Women (≥18 years old) with newly diagnosed International Federation of Gynecology and Obstetrics (FIGO) stage III EOC (serous, endometrioid, or mucinous) who underwent cytoreductive surgery up to 3 weeks prior to randomization and were scheduled for first-line platinum-based CT were eligible for this trial. Additional inclusion criteria were optimal debulking surgery (zero residuum or maximum residuum of <1 cm), and Eastern Cooperative Oncology Group performance status of 0–2. The major exclusion criteria included FIGO stage other than III, clear-cell histology, non-EOC/borderline tumor, residual disease with lesion(s) >1 cm, prior or current systemic anticancer therapy, prior or concurrent radiotherapy to the abdomen and pelvis, malignancy other than EOC (unless in complete remission for >3 years, or incidental low-grade carcinoid totally excised during primary debulking surgery without signs of metastasis), clinically significant comorbidities, known hypersensitivity to any constituent of DCVAC/OvCA, and systemic immunosuppressive therapy. The complete list of eligibility criteria is provided in the protocol (online supplemental material). All patients provided written informed consent.

10.1136/jitc-2021-003190.supp1 Supplementary data

### Trial design and treatments

Eligible patients were randomized at a 1:1:1 ratio using a centrally managed, interactive web-response system to one of three groups in Part 1: DCVAC/OvCa in parallel with CT (Group A), DCVAC/OvCa sequential to CT (Group B), or CT alone (Group C). All treatments were administered in an open-label manner to avoid exposing patients to unnecessary interventions, such as leukapheresis, which are generally deemed safe but may carry some inadvertent risk. After an interim analysis of Part 1, Part 2 was added as an expansion for Groups B and C to explore longer exposure to DCVAC/OvCa sequential to CT (see below for further details). In this part, patients were randomized at a 2:1 ratio to DCVAC/OvCa sequential to CT (Group B), or CT alone (Group C). This part was implemented in Protocol V.5.1. (August 4, 2017). In both parts, patients were stratified by the tumor residuum (0 or <1 cm).

Patients in Groups A and B were to undergo leukapheresis within 7 days after randomization. Patients in Group A received the first dose of DCVAC/OvCa at 2 weeks after the second dose of CT, and subsequent doses were administered 4±3 days before the next CT dose. After completion of CT, DCVAC/OvCa was administered every 6 weeks until all prepared doses had been used. In Group B, the first dose of DCVAC/OvCa was to be administered 2 weeks after completion of CT, with subsequent doses at 3-week intervals for the first five doses and then at 6-week intervals until all doses had been used. Ten doses of DCVAC/OvCa were prepared for Groups A and B in Part 1, and 15 doses were prepared for Group B in Part 2. Each dose of DCVAC/OvCa comprised approximately 10^7^ autologous DCs in 5 mL. The investigators could, at their discretion, continue treatment with DCVAC/OvCa available doses despite disease progression or after a change of the CT regimen.

All patients were to receive standard of care CT, comprizing paclitaxel at a dose of 175 mg/m^2^, intravenously over 3 hours, followed by carboplatin to achieve an area under the concentration-time curve of 5–7, intravenously over 30–60 min. Six cycles of CT were to be administered at 3-week intervals (±3 days), starting within 1 week after leukapheresis in Groups A and B or within 2 weeks of randomization in Group C.

An end-of-treatment visit was scheduled 30 days after the last dose of DCVAC/OvCa in Groups A and B, and 30 days after the last dose of CT in Group C. Patients were then followed-up every 6 weeks for 2 years after randomization for efficacy, and subsequently every 6 months for survival. Further details regarding the design of the trial and amendments are provided in the protocol (online supplemental materials). The final survival analysis is planned to take place 5 years after randomization of the last patient or once OS maturity reaches at least 50%, whichever occurs first.

### Preparation of DCVAC/OvCa

Each DCVAC/OvCa dose comprises DCs loaded with antigens derived from the EOC cell lines (OV-90 and SK-OV-3). To prepare DCVAC/OvCA, the peripheral blood mononuclear cells, obtained via leukapheresis and gradient centrifugation, are first cultured in a medium containing interleukin-4 and granulocyte-macrophage colony-stimulating factor. Immature DCs are separated, co-cultured (pulsed) with high hydrostatic pressure-treated OV-90 and SK-OV-3 cells, and matured using polyinosinic:polycytidylic acid.^11 12^ The resulting product is cryopreserved at a concentration of approximately 10^7^ DCs in 1 mL of CryoStor CS10 per vial.

### Endpoints and measurements

Safety was evaluated in terms of treatment-emergent adverse events (TEAEs), which were classified and graded according to the Common Terminology Criteria for Adverse Events V.4·03. Routine laboratory tests were also done before the start of each CT cycle and administration of DCVAC/OvCa. Additional laboratory tests and radiologic tumor assessments were permitted at the investigator’s discretion. The following TEAEs were considered of special interest: discontinuations due to a TEAE, systemic allergic reactions related to DCVAC/OvCa (other than local inflammatory reactions or irritation at the injection site), severe systemic infections related to DCVAC/OvCa, secondary malignancies, and symptomatic/clinically relevant autoimmune disorders.

The primary efficacy endpoint in this trial was PFS at 2 years after randomization. Secondary efficacy endpoints were remission rates at 6 months and 1 year, the biological progression-free interval (PFI_BIO_), time to first subsequent therapy (TFST), and OS. For protocol-relevant definitions of endpoints, please see the Statistical Analyses section.

Disease progression was determined by the investigator’s assessment according to modified Response Evaluation Criteria in Solid Tumours (RECIST) 1.1. Modified tumor response categories (ie, slow and non-slow progression) were defined for the purpose of the study. Non-slow progression was defined as a new non-nodal tumor lesion (diameter ≥20 mm on the long axis), new nodal lesion (pathological ≥15 mm), unequivocal progression of a tumor lesion present at screening but could not be measured (ie, tumor residuum), or unequivocal new tumor lesions that cannot be measured (eg, tumor residuum, bone lesions, mesenteric infiltration). Other cases of progression were classified as slow progression. Slow disease progression had to be confirmed by the next subsequent imaging using the same modality. In such cases, the date of progression was defined as the date of initial imaging. Histological or cytological confirmation of disease progression could substitute imaging in patients with peritoneal or pleural effusion. Patients with non-slow disease progression or those experiencing significant clinical deterioration could immediately start subsequent therapy, without a confirmatory imaging, if deemed necessary by the investigator. Patients with slow progression could continue their CT until progression was confirmed by the subsequent imaging.

CA-125 levels were measured to determine PFI_BIO_, which was defined as an increase in CA-125 levels at two separate measurements obtained at least 1 week apart, using the Gynecologic Cancer InterGroup definition.^18^ Imaging scans were performed and CA-125 levels were measured at weeks 10 (CT cycle 4), 18 (ie, 2 weeks after completing CT), 30, 42, 54, 68, 80, 92, and 104.

### Statistical analyses

The target was to randomize approximately 90 patients in Part 1 and 30 in Part 2. The safety analysis (SAF) set comprised patients who received at least one dose of CT or DCVAC/OvCa. Any patients in Group A or B who received at least one dose of CT, but did not start DCVAC/OvCa, were included in the SAF analysis set. Baseline characteristics and the primary and secondary efficacy endpoints were analyzed using the modified intention-to-treat (mITT) analysis set, which comprised all patients who received at least one dose of CT in Group C or at least one dose of DCVAC/OvCa for Groups A and B, allowing exploration of the effects of DCVAC/OvCa. Predefined sensitivity analyses of OS and further-line therapies were done using the intention-to-treat (ITT) analysis set, which comprised all randomized patients, regardless of whether they received any treatment or not.

All safety data were analyzed descriptively, and the relevant groups in both parts were pooled because there was no evidence for a difference in the safety profile between the original and prolonged exposure in Group B. PFS was defined as the time from randomization to the date of the first radiological progression or death, whichever came first. Remission was calculated at 6 months and 1 year after the last dose of CT. PFI_BIO_ was calculated as the time from randomization to biological disease progression, and a sensitivity analysis was done for patients regardless of whether biological disease progression was confirmed or not. The TFST was defined as the time from the randomization to the first dose of the subsequent therapy. OS was calculated as the time from randomization to death from any cause. Survival outcomes were plotted using the Kaplan-Meier method. Comparisons were made using unstratified log-rank tests. Efficacy data are mainly reported for Part 1 because Part 2 was limited in terms of the sample size and its exploratory intent. As an exploratory phase II trial, the trial was not powered to detect differences among the groups. All analyses were conducted using SAS software V.9.4 (SAS Institute).

### Role of the funding source

SOTIO a.s. was involved in trial design and conduct, data analyses, production of DCVAC/OvCa, and drafting of the manuscript.

## Results

### Patients

**Figure 1:**
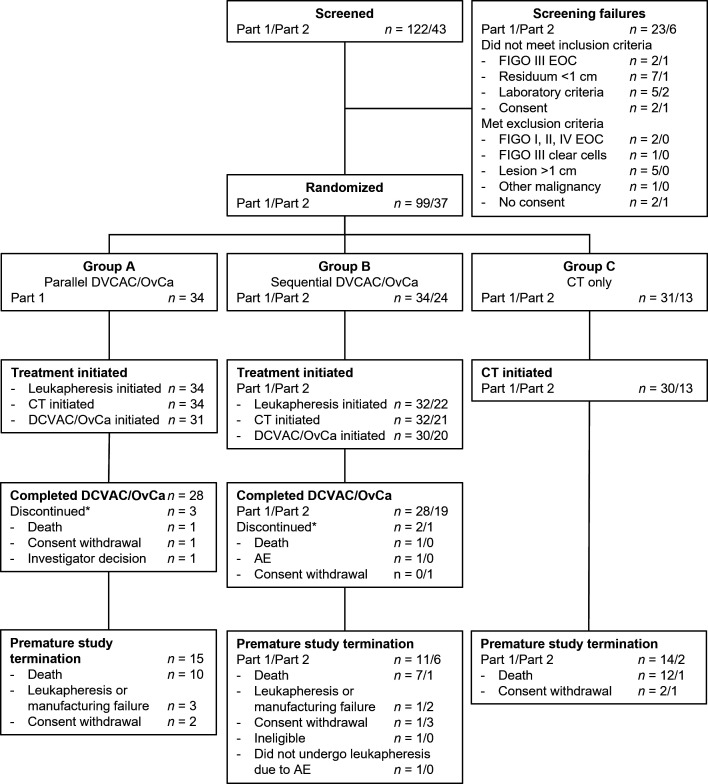
Patient disposition. *Discontinued DCVAC/OvCa. AE, adverse event; CT, chemotherapy; EOC, epithelial ovarian cancer; FIGO, International Federation of Gynecology and Obstetrics.

A total of 165 patients were initially screened across 15 sites. Of these, 99 were randomized across 13 sites in Czech Republic and Poland in Part 1 (Group A, n=34; Group B, n=34, Group C, n=31) and 37 patients in Czech Republic in Part 2 (Group B, n=24; Group C, n=13) (figure 1). DCVAC/OvCa was initiated in 31 patients in Group A and 30 patients in Group B in Part 1, and in 20 patients in Group B in Part 2. The mITT analysis set, which was used for the primary analyses of efficacy, comprised 90 patients in Part 1 and 33 in Part 2 (online supplemental table 1). The SAF analysis set comprised 96 and 34 patients in Parts 1 and 2, respectively.

The patient characteristics were generally well balanced between the treatment groups (table 1). The predominant histological type was serous ovarian cancer, classified as high grade. There was an apparent imbalance in the median tumor CD8 +cell count among the treatment groups, being lower in Group A in Part 1 than in the other groups (table 1).

**Table 1:** Patient characteristics (modified intention-to-treat analysis set)

Characteristic	Trial part 1			Trial part 2	
	Group A (N=31)	Group B (N=29)	Group C (N=30)	Group B (N=20)	Group C (N=13)
Age at randomization, years*	61.7 (24.2–72.7)	55.9 (20.7–73)	62.3 (46.4–74.5)	62.7 (32.9–70.7)	60.8 (43.2–72.6)
18–64 years	21 (67.7)	23 (79.3)	21 (70.0)	14 (70.0)	11 (84.6)
65–84 years	10 (32.3)	6 (20.7)	9 (30.0)	6 (30.0)	2 (15.4)
BMI, kg/m^2^†	24.7 (20.8–42.7)	26.3 (16.3–39.7)	25 (19.7–37.5)	25.1 (19.9–37.7)	24.8 (18.8–31.6)
Type of EOC					
Endometrioid	2 (6.5)	6 (20.7)	1 (3.3)	–	–
Mucinous	1 (3.2)	–	–	–	–
Serous	28 (90.3)	23 (79.3)	29 (96.7)	20 (100.0)	13 (100.0)
Time since diagnosis, days*	22 (14–105)	28 (9–95)	21.5 (12–113)	34 (16–145)	30 (15–40)
Time since primary debulking surgery, days*	22 (13–35)	22 (9–43)	21.5 (12–39)	31 (14–42)	29 (15–40)
Post surgery residual lesion, n	31	29	30	20	13
Residuum <1 cm	4 (12.9)	5 (17.2)	5 (16.7)	1 (5.0)	2 (15.4)
Zero residuum	27 (87.1)	24 (82.8)	25 (83.3)	19 (95.0)	11 (84.6)
*BRCA1* status,‡ n	16	12	12	10	8
Negative	11 (68.8)	8 (66.7)	7 (58.3)	8 (80.0)	6 (75.0)
Not available	–	–	–	1 (10.0)	–
Positive	5 (31.3)	4 (33.3)	5 (41.7)	1 (10.0)	2 (25.0)
*BRCA2* status,‡ n	15	10	12	10	7
Negative	14 (93.3)	9 (90.0)	9 (75.0)	9 (90.0)	5 (71.4)
Not available	–	–	1 (8.3)	1 (10.0)	–
Positive	1 (6.7)	1 (10.0)	2 (16.7)	–	2 (28.6)
Tumor CD8^+^ cell count,† n	29	23	26	18	13
Cells/mm^2^	40.4 (0.5–615.1)	110.5 (2.4–1092.4)	85.5 (1.9–376.9)	110.0 (20.2–494.7)	72.5 (2.3–600.1)

Values are median (range) or n (%).

*At randomization.

†At screening.

‡Assessment of *BRCA* status was not mandated in the protocol.

BMI, body mass index; EOC, epithelial ovarian cancer; Group A, DCVAC/OvCa in parallel with CT; Group B, CT and sequential DCVAC/OvCa; Group C, CT only.

In the SAF analysis set, the median number of doses of DCVAC/OvCa in Groups A and B was 10, with a median duration of exposure of 9.7 months (table 2). The median duration of exposure to CT was 3.5 months in Groups A, B, and C (table 2). All of the patients included in the SAF analysis set were exposed to CT (online supplemental table 2).

**Table 2:** Exposure to DCVAC/OvCa and CT for both trial periods combined (safety analysis set)

	Group A (N=34)	Group B (N=53)	Group C (N=43)
Number of DCVAC/OvCa doses administered			
n	34	53	–
Median (range)	10 (0–10)	10 (0–15)	–
Duration of exposure to DCVAC/OvCa, months			
n	31	50	–
Median (range)	9.7 (1.6–10)	9.7 (0.7–17)	
Number of CT doses administered			
n	34	53	43
Median (range)	12 (2–16)	12 (2–14)	12 (2–16)
Duration of exposure to CT, months			
n	34	53	43
Median (range)	3.5 (0–4.9)	3.5 (0–4.4)	3.5 (0–5.1)

CT, chemotherapy; Group B, CT and sequential DCVAC/OvCa; Group C, CT only; Gruop A, DCVAC/OvCa in parallel with CT.

### Safety

Safety data were combined for the corresponding groups in Parts 1 and 2. TEAEs occurred in 33 (97.1%), 51 (96.2%), and 37 (86.0%) patients in Groups A, B, and C, respectively (table 3). TEAEs resulted in death in 2 (3.8%) patients in Group B. The TEAEs resulting in death were pyelonephritis and retroperitoneal abscess in the first patient and pneumonia in the second patient. Serious TEAEs occurred in 27.9%–44.1% of patients, and Grade 3–5 TEAEs occurred in 55.8%–64.7% of patients.

**Table 3:** Overall safety for both trial periods combined (safety analysis set)

	Group A (N=34)	Group B (N=53)	Group C (N=43)
Any TEAEs	33 (97.1)	51 (96.2)	37 (86.0)
TEAEs leading to death	–	2 (3.8)	–
Serious TEAEs	15 (44.1)	18 (34.0)	12 (27.9)
Grade 3–5 TEAEs	22 (64.7)	32 (60.4)	24 (55.8)
TEAEs of special interest	–	2 (3.8)	–
DCVAC/OvCa-related TEAEs	2 (5.9)	2 (3.8)	–
TEAEs leading to discontinuation of DCVAC/OvCa	–	1 (1.9)	–
Leukapheresis-related AEs	5 (14.7)	3 (5.7)	–
CT-related TEAEs	29 (85.3)	50 (94.3)	35 (81.4)
TEAEs leading to discontinuation of CT	1 (2.9)	1 (1.9)	1 (2.3)

Values are n (%)

CT, chemotherapy; Group A, DCVAC/OvCa in parallel with CT; Group B, CT and sequential DCVAC/OvCa; Group C, CT only; TEAE, treatment-emergent adverse event.

TEAEs that were considered by the investigators to be related to DCVAC/OvCa occurred in two patients in Group A (inflammation of the left axilla and facial erythema) and two patients in Group B (injection site erythema and injection site pain in one patient; drug hypersensitivity in one patient). The event of drug hypersensitivity resulted in discontinuation of DCVAC/OvCa. Drug hypersensitivity, which was considered a TEAE of special interest, occurred two times in this patient at an interval of 19 days. Although both events recovered/resolved, DCVA/OvCa was discontinued following the second event.

Breast cancer was recorded as a TEAE of special interest in one patient in Group B. This event was classified as Grade 3, and serious, and was detected 336 days after starting treatment.

Five patients (14.7%) in Group A and three patients (5.7%) in Group B experienced TEAEs related to leukapheresis. These events were post-procedural hematoma, decreased hemoglobin, hypocalcemia, dermatitis allergic, and hypotension in Group A, and procedural nausea, procedural pain, tachycardia, and paresthesia in Group B. None of these events led to discontinuation of leukapheresis.

TEAEs related to CT occurred in over 80% of patients, and resulted in discontinuation of CT in one patient in each group. The most common TEAEs (≥30% of patients in any group) were neutropenia, anemia, nausea, and thrombocytopenia, all of which are commonly associated with CT (table 4); likewise, the most common Grade 3–5 TEAEs were known side effects of CT.

**Table 4:** TEAEs in >10% of patients in any group, Grade 3–5 TEAEs in >5% of patients in any group, and TEAEs related to DCVAC/OvCa for both trial periods combined

Preferred term	Group A (N=34)	Group B (N=53)	Group C (N=43)
TEAEs related to DCVAC/OvCa			–
Inflammation	1 (2.9)	–	–
Erythema	1 (2.9)	–	–
Injection site erythema	–	1 (1.9)	–
Injection site pain	–	1 (1.9)	–
Drug hypersensitivity	–	1 (1.9)	–
TEAEs in >10% of patients			
Neutropenia	18 (52.9)	22 (41.5)	21 (48.8)
Anemia	13 (38.2)	22 (41.5)	19 (44.2)
Nausea	10 (29.4)	18 (34.0)	9 (20.9)
Thrombocytopenia	8 (23.5)	16 (30.2)	13 (30.2)
Paresthesia	8 (23.5)	15 (28.3)	11 (25.6)
Leukopenia	8 (23.5)	8 (15.1)	6 (14.0)
Arthralgia	5 (14.7)	13 (24.5)	4 (9.3)
Fatigue	5 (14.7)	9 (17.0)	4 (9.3)
Vomiting	8 (23.5)	3 (5.7)	4 (9.3)
Neuropathy peripheral	4 (11.8)	7 (13.2)	4 (9.3)
Fever	3 (8.8)	6 (11.3)	5 (11.6)
Constipation	3 (8.8)	5 (9.4)	6 (14.0)
Alopecia	3 (8.8)	8 (15.1)	2 (4.7)
Drug hypersensitivity	–	7 (13.2)	5 (11.6)
Hypokalemia	5 (14.7)	4 (7.5)	2 (4.7)
Urinary tract infection	5 (14.7)	4 (7.5)	2 (4.7)
Hypersensitivity	2 (5.9)	6 (11.3)	2 (4.7)
Nasopharyngitis	2 (5.9)	6 (11.3)	2 (4.7)
Weight decreased	1 (2.9)	4 (7.5)	5 (11.6)
Insomnia	1 (2.9)	6 (11.3)	–
Grade 3–5 TEAEs in >5% of patients			
Neutropenia	10 (29.4)	17 (32.1)	14 (32.6)
Anemia	5 (14.7)	7 (13.2)	6 (14.0)
Thrombocytopenia	4 (11.8)	5 (9.4)	8 (18.6)
Leukopenia	2 (5.9)	6 (11.3)	2 (4.7)
Vomiting	3 (8.8)	–	1 (2.3)
Infected lymphocele	2 (5.9)	2 (3.8)	–
Pancytopenia	2 (5.9)	–	–

Values are n (%)

CT, chemotherapy; Group A, DCVAC/OvCa in parallel with CT; Group B, CT and sequential DCVAC/OvCa; Group C, CT only; TEAE, treatment-emergent adverse event.

There were 14 deaths in Group A, 11 in Group B, and 15 in Group C, of which 12, 9, and 14, respectively, were considered related to disease progression. For the other five patients, the cause of death was unknown for two patients in Group A, a TEAE for two patients in Group B (as described above), and meningoencephalitis viral for one patient in Group C. The latter event was not considered a TEAE.

### PFS

**Figure 2:**
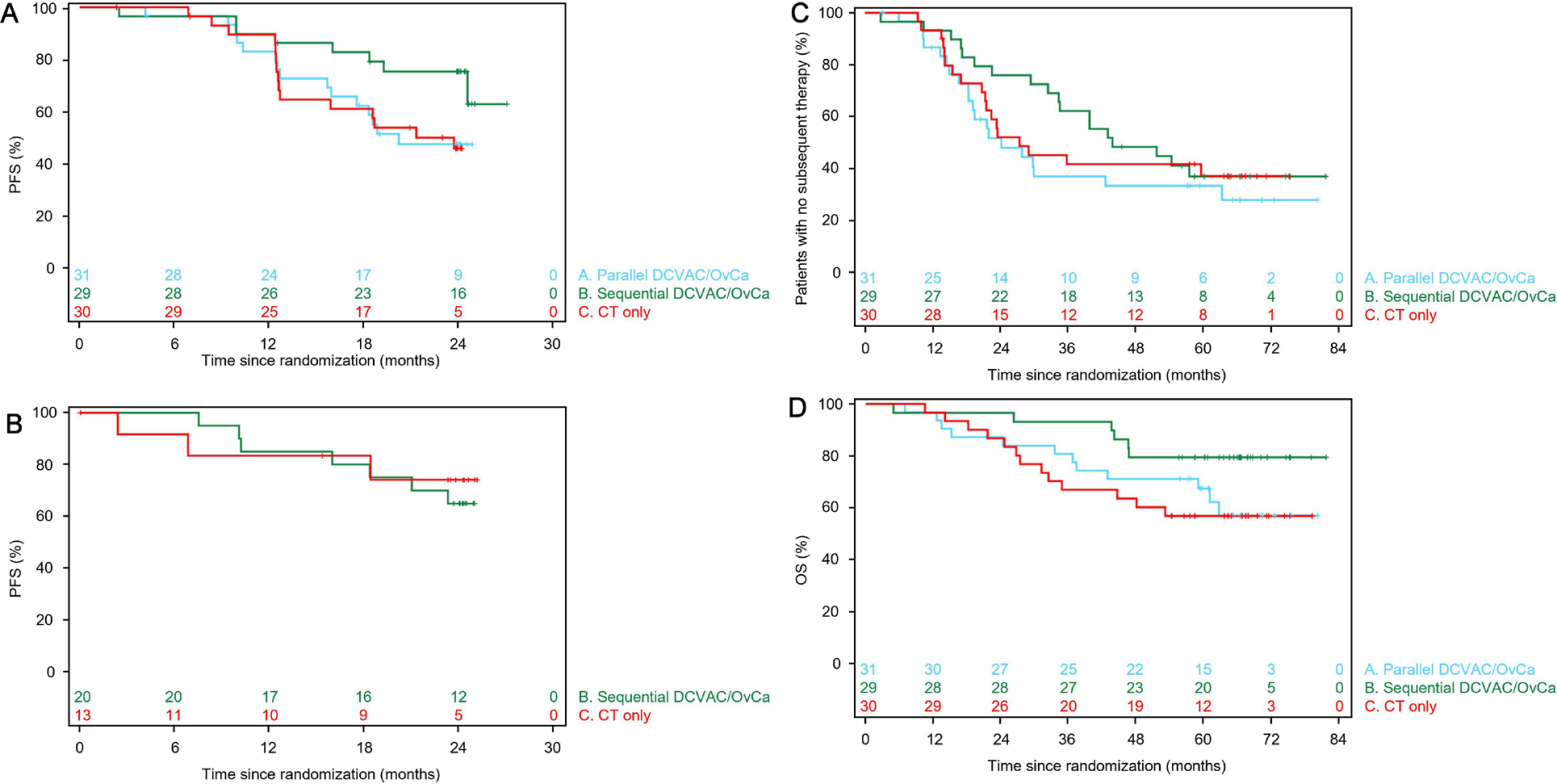
Kaplan-Meier plots of progression-free survival in Parts 1 (A) and Part 2 (B), patients without subsequent therapy in Part 1 (C), and overall survival in Part 1 (D). Plots are shown for the modified intention-to-treat analysis set. CT, chemotherapy; Group A, DCVAC/OvCa in parallel with CT; Group B, CT and sequential DCVAC/OvCa; Group C, CT only; OS, overall survival; PFS, progression-free survival.

Due to data immaturity and the small sample size in Part 2, the efficacy analyses are presented for Part 1 only, with the exception of PFS. The Kaplan-Meier plots of PFS for the mITT in Part 1 are shown in figure 2A. With a data maturity of 42.2%, the Kaplan-Meier estimate of PFS at 2 years (95% CI) was 47% (28% to 64%) in Group A, 75% (55% to 87%) in Group B, and 46% (27% to 63%) in Group C. The HR (95% CI) for PFS in Group A versus Group C was 0.98 (0.48 to 2.00; p=0.9483) and that in Group B versus Group C was 0.39 (0.16 to 0.96; p=0.0336) (table 5). Median PFS was 20.3 months in Group A, not reached in Group B, and 21.4 months in Group C. These data indicate that sequential DCVAC/OvCa was associated with a reduced risk of disease progression, but there was no significant benefit of DCVAC/OvCa when administered in parallel with CT in Part 1. As an exploratory analysis, we also determined PFS in Part 2 (figure 2B). However, at the time of the data export, 7 of 20 patients (35.0%) in Group B and 3 of 13 (23.1%) in Group C had experienced progression, resulting in a data maturity rate of 30.3% (table 5). Median PFS had not been reached in either group.

**Table 5:** Efficacy outcomes in Parts 1 and 2 (modified intention-to-treat analysis set)

Outcome		Parameter	Trial part 1			Trial part 2	
			Group A (N=31)	Group B (N=29)	Group C (N=30)	Group B (N=20)	Group C (N=13)
PFS	Disease progression	n (%)	15 (48.4)	8 (27.6)	15 (50.0)	7 (35.0)	3 (23.1)
	Time to event, months	Median (95% CI)	20.3 (15.7 to NA)	NA (24.6 to NA)	21.4 (12.6 to NA)	NA (18.4 to NA)	NA (6.9 to NA)
	At 1 year	KM (95% CI)	83% (64% to 92%)	90% (71% to 97%)	89% (71% to 96%)	85% (60% to 95%)	83% (48% to 96%)
	At 2 years	KM (95% CI)	47% (28% to 64%)	75% (55% to 87%)	46% (27% to 63%)	65% (40% to 82%)	74% (39% to 91%)
	Data maturity	Total events (%)	38 (42.2)	–	–	10 (30.3)	
	HR vs Group C (95% CI)		0.98 (0.48 to 2.00)	0.39 (0.16 to 0.96)	–	1.29 (0.33 to 4.99)	–
	Log-rank p value		0.9483	0.0336	–	0.7125	–
PFI_BIO_	Biological progression	n (%)	2 (6.5)	1 (3.4)	2 (6.7)	–	–
	Time to event, months	Median (95% CI)	NA (NA to NA)	NA (NA to NA)	NA (NA to NA)	–	–
	At 1 year	KM (95% CI)	100% (100% to 100%)	100% (100% to 100%)	100% (100% to 100%)	–	–
	At 2 years	KM (95% CI)	91% (68% to 98%)	96% (74% to 99%)	89% (63% to 97%)	–	–
	Data maturity	Total events (%)	5 (5.6)	–	–	–	–
	HR vs Group C (95% CI)		0.89 (0.13 to 6.31)	0.35 (0.03 to 3.87)	–	–	–
	Log-rank p value		0.9055	0.3704	–	–	–
PFI_BIO_	Biological progression	n ()	10 (32.3)	4 (13.8)	7 (23.3)	–	–
	Time to event, months	Median (95% CI)	24.4 (24.1 to NA)	NA (NA to NA)	NA (NA to NA)	–	–
	At 1 year	KM (95% CI)	86% (67% to 95%)	93% (74% to 98%)	93% (74% to 98%)	–	–
	At 2 years	KM (95% CI)	71% (51% to 85%)	84% (63% to 94%)	72% (50% to 86%)	–	–
	Data maturity	Total events (%)	21 (23.3)	–	–	–	–
	HR vs Group C (95% CI)		1.14 (0.42 to 3.08)	0.51 (0.15 to 1.74)	–	–	–
	Log-rank p value		0.7963	0.2723	–	–	–
Remission	At 6 months	n (%)	25 (80.6)	28 (96.6)	25 (83.3)	–	–
	At 1 year	n (%)	20 (64.5)	23 (79.3)	17 (56.7)	–	–
OS	Deaths	n (%)	12 (38.7)	6 (20.7)	13 (43.3)	–	–
	Time to event, months	Median (95% CI)	NA (59 to NA)	NA (NA to NA)	NA (34.9 to NA)	–	–
	At 1 year	KM (95% CI)	97% (79% to 100%)	97% (78% to 100%)	97% (79% to 100%)	–	–
	At 2 years	KM (95% CI)	87% (69% to 95%)	97% (78% to 100%)	87% (68% to 95%)	–	–
	At 3 years	KM (95% CI)	81% (62% to 91%)	93% (75% to 98%)	67% (47% to 80%)	–	–
	At 4 years	KM (95% CI)	71% (52% to 84%)	79% (60% to 90%)	63% (44% to 78%)	–	–
	At 5 years	KM (95% CI)	67% (48% to 81%)	79% (60% to 90%)	57% (37% to 72%)	–	–
	Data maturity	Total events (%)	31 (34.4)	–	–	–	–
	HR vs Group C (95% CI)		0.84 (0.38 to 1.84)	0.40 (0.15 to 1.06)	–	–	–
	Log-rank p value		0.6631	0.0557	–	–	–

Group A, DCVAC/OvCa in parallel with chemotherapy (CT); Group B, CT and sequential DCVAC/OvCa; Group C, CT only; KM, Kaplan-Meier; OS, overall survival; PFI_BIO_, biological progression-free interval; PFS, progression-free survival.

### Biological progression and disease remission

The median PFI_BIO_ was not reached in any group in Part 1 (table 3). The Kaplan-Meier estimates of PFI_BIO_ at 2 years were ≥89% in each group, but data maturity was low (5.6%). The 1-year remission rates in Part 1 were 64.5%, 79.3%, and 56.7% in Groups A, B and C, respectively (table 5).

### Time to first subsequent therapy

The Kaplan-Meier plots of initiation of first subsequent therapy in Groups A–C in Part 1 are shown in figure 2C. Although the proportions of patients who started a subsequent therapy were similar in all three groups, TFST was longest in Group B (online supplemental table 3). However, the HR for Group B versus Group C did not reach statistical significance (0.83; 95% CI 0.43 to 1.60).

### OS

The Kaplan-Meier plots suggested an OS benefit in Group B in Part 1, with estimated OS at 4 years of 71%, 79%, and 63% in Groups A, B and C, respectively (figure 2D, table 5). Median OS was not reached in any group and the OS data were immature. The OS data were consistent across the mITT (table 5) and ITT analysis sets (online supplemental table 4).

## Discussion

This trial was performed to explore the safety and efficacy signals of DCVAC/OvCa. We investigated two distinct schedules of DCVAC/OvCa administration: parallel and sequential. First, we explored the synergic effect of concomitant administration of DCVAC/OvCa and CT (paclitaxel plus carboplatin) and second we explored the advantage of reducing the tumor mass by prior CT in the maintenance setting, potentially allowing for a quicker onset of the clinical effect of DCVAC/OvCa immunotherapy.

Regarding safety, we found that TEAEs related to DCVAC/OvCa were rare, occurring in four patients, and resulted in the discontinuation of DCVAC/OvCa in only one patient. Furthermore, the vast majority of TEAEs recorded in this trial were expected effects of CT and considered related to CT by the investigator.

From an efficacy perspective, the trial indicated a significant improvement in PFS (primary efficacy endpoint of Part 1) in patients who were treated with sequentially administered DCVAC/OvCa following CT (ie, Group B) with a 61% lower risk of progression versus CT alone (ie, Group C). The significant difference in PFS was reached even though the trial was not specifically powered for comparisons of efficacy outcomes between the groups. This improvement in PFS was accompanied by a trend for an OS benefit (p=0.0557), with a 60% lower risk of death versus CT alone. Moreover, the TFST was longer in Group B, with a median of 43.9 months as compared with 27.4 months in Group C. Nevertheless, approximately two-thirds of patients in each group started a subsequent therapy during the trial. Thus, DCVAC/OvCa appears to induce a durable immune response and stabilize the disease, to extend survival in patients with ovarian cancer.

Part 2 was performed as an expansion for Groups B and C, with the intention to support hypothesis generation. The sample size in this part was very limited and the data were immature, as demonstrated by the analysis of the primary endpoint. Thus, further analyses of efficacy were not done. Future analyses of OS are planned at 5 years after randomization of the last patient or once data maturity is reached, whichever comes first.

Prior studies have demonstrated a prognostic role of CD8^+^ cells in patients with ovarian cancer.^19–22^ Immunohistochemical analyses of tumor samples of patients in the mITT analysis set revealed an imbalance in the density of tumor CD8^+^ cells, which was much lower in Group A than in Groups B and C. The different immunological malignant tissue profiles may translate into worse disease outcomes in Group A than Group B. DC-based immunotherapy as monotherapy generally fails to sufficiently reverse tumor progression, leading to the development of combination regimens. Thus, an appropriate combination of tumor mass reduction (cytoreduction by surgery, administration of cytotoxic agents, and/or radiotherapy) accompanied by robust immunomodulation might provide suitable conditions for inducing an antitumor immune response by the active immunotherapy.^23 24^ In EOC, carboplatin has been shown to improve the function of immune effector cells, including DCs and CD8^+^ T cells, and induced so-called ‘immunogenic cell death’, which is associated with the delivery of multiple adjuvant-like signals for DCs.^23^ Preclinical and clinical findings indicate that carboplatin may support the functions of DCVAC/OvCa, supporting an adaptive immune response with therapeutic potential. Further analyses are planned to investigate whether immune biomarkers are associated with the prognosis of patients treated with DCVAC/OvCa and better understand whether the underlying immunological capacity contributes to the possible differential effects between parallel and sequential administration of DCVAC/OvCa.

To date, several immunotherapy strategies have been studied in EOC, but most trials have focused on advanced or relapsed cancer.^9 25–33^ The data obtained in the present study provide evidence supporting continued development of advanced cellular based immunotherapies strategies for EOC in first-line treatment.

The present trial has some limitations. First, the trial was not powered for PFS and OS, so the possibilities for exploring these outcomes are limited. Second, imaging scans were not performed to assess disease progression in patients who dropped out from the trial prematurely. The ITT analysis set included several patients who dropped out early in the trial and lacked post-baseline tumor assessments, so their contribution to PFS is limited. Their results did not yield any new information and are not presented. In addition, patients in Group B would only be included in the mITT population if they did not progress during CT, which might not necessarily be the case for Groups A and C.

### Conclusions

In conclusion, DCVAC/OvCa (and leukapheresis) was not associated with significant safety concerns because most TEAEs observed in this trial were known side effects of CT. A statistically significant effect of DCVAC/OvCA administered sequentially to CT on PFS was observed in patients undergoing first-line treatment of EOC. Compared with CT alone, DCVAC/OvCa sequential to CT delayed disease progression, and showed trends towards extended TFST and OS. Larger, well-designed trials may be required to fully explore the potential of DCVAC/OvCa in EOC.

## Data Availability

Data are available upon reasonable request. The data sets generated during and/or analyzed during the trial are not publicly available due to commercial requirements, but are available from the corresponding author on reasonable request.
