# Supplementary material for: Safety and efficacy of dendritic cell-based immunotherapy DCVAC/OvCa added to first-line chemotherapy (carboplatin plus paclitaxel) for epithelial ovarian cancer: a phase 2, open-label, multicenter, randomized trial
Source: J Immunother Cancer. 2022 Jan 6;10(1):e003190. doi: 10.1136/jitc-2021-003190 (PMC8739446; doi:10.1136/jitc-2021-003190)
Supplement: Supplementary data [file jitc-2021-003190supp001.pdf]

Safety and efficacy of dendritic cell-based immunotherapy DCVAC/OvCa added to first-line chemotherapy (carboplatin plus paclitaxel) for epithelial ovarian cancer: A Phase 2, open-label, multicenter, randomized trial

SUPPLEMENTARY MATERIALS

Contents **Page**

Participating institutions 2
Members of the steering committee and data monitoring committee 2
Supplementary table 1. Numbers of patients per group in each analysis set 3
Supplementary table 2. Distribution of CT doses for both trial periods combined (SAF analysis set) 4
Supplementary table 3. Initiation of subsequent therapy in Part 1 (mITT and ITT analysis sets) 5
Supplementary table 4. Overall survival outcomes in Part 1 (intention-to-treat analysis set) 6

Participating institutions

Czech Republic: University Hospital Motol, Prague (Marek Pluta), University Hospital Brno, Brno (Lubos Minar), General University Hospital, Prague (David Cibula), University Hospital Olomouc, Olomouc (Bohuslav Melichar), University Hospital Hradec Kralove, Hradec Kralove (Jiri Spacek), Hospital Ceske Budejovice, Ceske Budejovice (Petr Valha), University Hospital Ostrava, Ostrava (Jaroslav Klat), Masaryk Memorial Cancer Institute, Brno (Josef Chovanec), Hospital Novy Jicin a.s., Novy Jicin (Pavel Bartos), University Hospital Plzen, Plzen (Zdenek Novotny), University Hospital Kralovske Vinohrady, Prague (Lukas Rob).
Germany: Universitaets - Frauenklinik Koeln, Koeln (Peter Mallmann); Universitaetsklinikum Frauenklinik Erlangen, Erlangen (Alexander Hein).
Poland: Uniwersytecki Szpital Kliniczny v Bialymstoku, Bialystok (Pawel Knapp), I Oddzial Ginekologii Onkologicznej, Lublin (Dariusz Kieszko).

Members of the steering committee and data monitoring committee

Steering Committee: Lukas Rob (Third Faculty of Medicine, Charles University and University Hospital Kralovske Vinohrady, Prague, Czech Republic), David Cibula (First Faculty of Medicine, Charles University and General University Hospital in Prague, Prague, Czech Republic), Peter Mallmann (University Hospital of Cologne, Cologne, Germany) and Pawel Knapp (Department of Gynaecologic Oncology, Medical University of Bialystok, Bialystok, Poland).

Independent Data Monitoring Committee: Krzystof Gawrychowski (Department of Gynecological Oncology and Oncology, Medicover Hospital, Warsaw, Poland), Philipp Harter (Department of Gynecology and Gynecologic Oncology, Ev. Kliniken Essen-Mitte, Essen, Germany), and Rebecca Kristeleit (University College London Cancer Institute, London, United Kingdom).

Supplementary tables

Supplementary table 1. Numbers of patients per group in each analysis set

**Analysis sets**	**Trial part 1**			**Trial part 2**	
	**Group A**	**Group B**	**Group C**	**Group B**	**Group C**
Randomized	34	34	31	24	13
ITT	34	32	31	24	13
Excluded (leukapheresis not initiated)	-	2 (6.3%)	-	-	-
mITT (primary efficacy analysis set)	31	29	30	20	13
Excluded	3 (9.7%)	5 (17.2%)	1 (3.3%)	4 (20.0%)	-
Did not receive at least one dose of DCVAC/OvCa (Groups A and B)	3 (100.0%)	4 (80.0%)	-	4 (100.0%)	-
Did not receive at least one dose of CT (Group C)	-	-	1 (100.0%)	-	-
Protocol deviation (ineligible)	-	1 (20.0%)	-	-	-
LEU	34	32	NA	22	NA
Excluded	-	2 (6.3%)	NA	2 (9.1%)	NA
Leukapheresis not initiated	-	2 (100.0%)	NA	2 (100.0%)	NA
SAF	34	32	30	21	13
Excluded from SAF	-	2 (3.6%)	1 (3.3%)	3 (14.3%)	-
Not administered at least one dose of CT or at least one dose of DCVAC/OvCa	-	2 (100.0%)	1 (100.0%)	3 (100.0%)	-

Values are *n* or *n* (%)

Group A, DCVAC/OvCa in parallel with chemotherapy (CT); Group B, CT and sequential DCVAC/OvCa; Group C, CT only; ITT, intention-to-treat; mITT, modified intention-to-treat; LEU, leukapheresis; SAF, safety analysis

Supplementary table 2. Distribution of CT doses for both trial periods combined (SAF analysis set)

	**Group A (*N* = 34)**	**Group B (*N* = 53)**	**Group C (*N* = 43)**
Number of CT doses			
2	3 (8.8%)	2 (3.8%)	1 (2.3%)
6	-	2 (3.8%)	-
8	1 (2.9%)	1 (1.9%)	-
10	2 (5.9%)	1 (1.9%)	3 (7.0%)
11	-	1 (1.9%)	-
12	25 (73.5%)	43 (81.1%)	36 (83.7%)
14	2 (5.9%)	3 (5.7%)	2 (4.7%)
16	1 (2.9%)	-	1 (2.3%)
Number of doses of cisplatin*			
1	-	1 (1.9%)	-
2	-	1 (1.9%)	1 (2.3%)
4	-	1 (1.9%)	-
Number of doses of cyclophosphamide*			
7	-	1 (1.9%)	-
Number of doses of doxorubicin*			
2	-	1 (1.9%)	-

*First-line agent not recommended in the protocol, reported as protocol deviations
Values are *n* (%)
CT, chemotherapy; SAF, safety analysis; Group A, DCVAC/OvCa in parallel with CT; Group B, CT and sequential DCVAC/OvCa; Group C, CT only

Supplementary table 3. Initiation of subsequent therapy in Part 1 (mITT and ITT analysis sets)

Analysis set	Outcome	Group A	Group B	Group C
mITT	*N*	31	29	30
	Subsequent therapy initiated, *n* (%)	20 (64.5%)	18 (62.1%)	18 (60.0%)
	TFST, median (95% CI)	24.1 (18.3–63.3)	43.9 (32.5–NA)	27.4 (20.7–NA)
	KM estimates for not starting subsequent therapy (95% CI)			
	At 1 year	87% (68%–95%)	93% (75%–98%)	93% (76%–98%)
	At 2 years	52% (32%–68%)	76% (56%–88%)	52% (33%–68%)
	At 3 years	37% (20%–54%)	62% (42%–77%)	42% (24%–58%)
	At 4 years	33% (17%–50%)	48% (29%–65%)	42% (24%–58%)
	At 5 years	33% (17%–50%)	37% (20%–54%)	37% (20%–54%)
	Data maturity, total events (%)	56 (62.2%)		
	HR vs Group C (95% CI)	1.24 (0.66–2.35)	0.83 (0.43–1.60)	
	Log-rank p-value	0.5085	0.5785	
ITT	*N*	34	32	31
	Subsequent therapy initiated, n (%)	21 (61.8%)	18 (56.3%)	18 (58.1%)
	TFST, median (95% CI)	24.1 (18.3–63.3)	43.9 (32.5–NA)	29.0 (21.4–NA)
	KM estimates for not starting subsequent therapy (95% CI)			
	At 1 year	84% (66%–93%)	93% (75%–98%)	94% (77%–98%)
	At 2 years	52% (33%–67%)	76% (56%–88%)	54% (34%–69%)
	At 3 years	38% (21%–55%)	62% (42%–77%)	43% (26%–60%)
	At 4 years	34% (18%–51%)	48% (30%–65%)	43% (26%–60%)
	At 5 years	34% (18%–51%)	37% (20%–54%)	39% (22%–56%)
	Data maturity, total events (%)	57 (58.8%)		
	HR vs Group C (95% CI)	1.28 (0.68–2.41)	0.88 (0.46–1.69)	
	Log-rank p-value	0.4367	0.7017	

CT, chemotherapy; mITT, modified intention-to-treat; ITT, intention-to-treat; Group A, DCVAC/OvCa in parallel with CT; Group B, CT and sequential DCVAC/OvCa; Group C, CT only; TFST, time to first subsequent therapy; CI, confidence interval; HR, hazard ratio

Supplementary table 4. Overall survival outcomes in Part 1 (intention-to-treat analysis set)

Outcome	Parameter	Group A	Group B	Group C
N		34	32	31
Deaths	*n* (%)	14 (41.2%)	9 (28.1%)	13 (41.9%)
Time to event	Median (95% CI)	NA (42.9–NA)	NA (NA–NA)	NA (34.9–NA)
At 1 year	KM (95% CI)	94% (78%–98%)	94% (77%–98%)	97% (79%–100%)
At 2 years	KM (95% CI)	82% (65%–92%)	91% (74%–97%)	87% (69%–95%)
At 3 years	KM (95% CI)	76% (58%–87%)	88% (70%–95%)	68% (48%–81%)
At 4 years	KM (95% CI)	68% (49%–81%)	75% (56%–87%)	65% (45%–79%)
At 5 years	KM (95% CI)	64% (46%–78%)	75% (56%–87%)	58% (39%–73%)
Data maturity	Total events (%)	36 (37.1%)		
HR vs group C (95% CI)		0.97 (0.46–2.07)	0.60 (0.25–1.40)	-
Log-rank p-value		0.9429	0.2279	-

ITT, intention-to-treat; Group A, DCVAC/OvCa in parallel with CT; Group B, CT and sequential DCVAC/OvCa; Group C, CT only; PFS, progression-free survival; CI, confidence interval; HR, hazard ratio; PFI_{BIO}, biological progression-free interval; OS, overall survival

Rob L, *et al. J Immunother Cancer* 2022; 10:e003190. doi: 10.1136/jitc-2021-003190
